# Modeling biomedical experimental processes with OBI

**DOI:** 10.1186/2041-1480-1-S1-S7

**Published:** 2010-06-22

**Authors:** Ryan R Brinkman, Mélanie Courtot, Dirk Derom, Jennifer M Fostel, Yongqun He, Phillip Lord, James Malone, Helen Parkinson, Bjoern Peters, Philippe Rocca-Serra, Alan Ruttenberg, Susanna-Assunta Sansone, Larisa N Soldatova, Christian J Stoeckert, Jessica A Turner, Jie Zheng, the OBI consortium

**Affiliations:** 1 British Columbia Cancer Agency, Vancouver, Canada; 2 Victoria University of Wellington, New Zealand; 3 Global Health Sector, SRA International, Inc, Durham, NC, USA; 4 University of Michigan Medical School, Ann Arbor, USA; 5 School of Computing Science, Newcastle University, UK; 6 The European Bioinformatics Institute, Cambridge, UK; 7 La Jolla Institute for Allergy and Immunology, La Jolla, CA, USA; 8 Science Commons, Cambridge, MA, USA; 9 Aberystwyth University, Wales, UK; 10 Center for Bioinformatics, Department of Genetics, University of Pennsylvania School of Medicine, Philadelphia, PA, USA; 11 Department of Psychiatry and Human Behavior, University of California, Irvine, CA, USA

## Abstract

**Background:**

Experimental descriptions are typically stored as free text without using standardized terminology, creating challenges in comparison, reproduction and analysis. These difficulties impose limitations on data exchange and information retrieval.

**Results:**

The Ontology for Biomedical Investigations (OBI), developed as a global, cross-community effort, provides a resource that represents biomedical investigations in an explicit and integrative framework. Here we detail three real-world applications of OBI, provide detailed modeling information and explain how to use OBI.

**Conclusion:**

We demonstrate how OBI can be applied to different biomedical investigations to both facilitate interpretation of the experimental process and increase the computational processing and integration within the Semantic Web. The logical definitions of the entities involved allow computers to unambiguously understand and integrate different biological experimental processes and their relevant components.

**Availability:**

OBI is available at http://purl.obolibrary.org/obo/obi/2009-11-02/obi.owl

## Background

Biomedical investigations use empirical approaches to investigate causal relationships among a large range of variables. The wide range of possible investigations presents a number of challenges when building tools to describe experimental processes. There are varying levels of complexity and granularity and a wide range of material and equipment is used. Furthermore, the use of varying terminology by different communities makes data integration problematic when representing and integrating biomedical investigations across different fields of study. The use of ontologies has been successful in biological data integration and representation [1,2] and there have been multiple efforts to develop ontologies aimed at providing clearer semantics for data (GO, FuGO, MGED, EXPO, LABORS, MSI ontology) [3-8]. Work in the transcriptomics, proteomics and metabolomics communities has proceeded in parallel, producing ontologies with overlapping scopes.

Though each focuses on particular types of experimental processes, many terms, such as investigation and assay, are common to all. Merging common aspects of these formalisms is useful as it provides a mechanism by which terms can be used and understood by all, reducing ambiguity and difficulties associated with post-hoc attempts to integrate data. The practice of consolidating representations is endorsed by organizations such as the OBO Foundry [9] which requires all member ontologies to define a term only once among them (orthogonality). OBO Foundry members use a common set of relations from the Relations Ontology [10], and the upper level Basic Formal Ontology (BFO) [11] in order to facilitate cross ontology consistency and to support automated reasoning [9]. OBO ontologies adhere to common naming conventions [12] in order to make it easier to learn and understand them.

The Ontology for Biomedical Investigations (OBI) addresses the need for a cross-disciplinary, integrated ontology for the detailed description of biological and clinical investigations. OBI is collaboratively developed by representatives from 19 biomedical communities from around the globe and has been submitted as a candidate for the OBO Foundry [9]. It uses other OBO ontologies wherever possible. OBI defines a set of broadly applicable terms that span biomedical and technological domains, for example, assay (the planned process of producing data about something) as well as domain-specific terms relevant to smaller areas of study, for example, T cell epitope recognition assay, used by the IEDB database to describe experimental data extracted from articles investigating immune epitopes [13].

OBI represents all phases of experimental processes, and the entities involved in preparing for, executing, and interpreting those processes *e.g.*, study designs, protocols, instrumentation, biological material, collected data and analyses performed on that data. OBI also represents roles and functions used in biomedical investigations. OBI therefore supports consistent annotation of biomedical experimental processes regardless of the field of study. OBI is expressed in OWL, a W3C ontology language developed for the Semantic Web. The development of OBI is driven by specific use cases of experiments. In this paper, the OBI release of 2009-11-02 is applied to three exemplar use cases, originating from three communities: 1) neuroscience, 2) vaccine protection, and 3) functional genomics. The OBI release of 2009-11-02 is available at http://purl.obolibrary.org/obo/obi/2009-11-02/obi.owl.

## Results

In what follows, *italics* are used to refer to a specific term within OBI where appropriate. OBI defines an *investigation* as a *process* with several parts, including planning an overall *study design*, executing the designed study, and documenting the results. An *investigation* typically includes *interpreting data* to draw conclusions.

Biomedical experimental processes involve numerous sub-processes, involving experimental materials such as whole organisms, organ sections and cell cultures. These experimental materials are represented as subclasses of the BFO class *material entity*. OBI uses BFO’s *material entity* as the basis for defining physical things. *Material entity* is an *independent continuant*, a continuant that is a bearer of quality and realizable entity(s), in which other entities inhere and which itself cannot inhere in anything [11]. *Material entities* are entities that are spatially extended, whose identity is independent of that of other entities, and which persist through time, for example *organism*, *test tube*, and *centrifuge*. *Material entities* can bear *roles*, typically socially defined, which are realized in the context of a *process*, e.g. *study subject role*, *host role*, *specimen role*, *patient role*; and *functions,* results of design or evolution that depend on their physical structure e.g. *measure function*, *separation function* and *environment control function*. The *function* is considered to *inhere in* the *material entity* and *be realized by* the role that material entity plays in a process.

To assess the completeness of the OBI release of 2009-11-02 and to demonstrate the use of OBI for annotation, we present three representative use cases. These demonstrate how to model entities and relations between entities involved in experimental processes using OBI. The first use case models a neuroscience experiment described in a journal article [14] and shows how logical definitions are constructed using parts of external ontologies imported into OBI. The second use case details how OBI is used to model vaccine studies; the third describes an investigation run by a Robot Scientist which fully automatically designs and executes functional genomics experiments.

## Use case 1: neuroscience investigation

**Figure 1:**
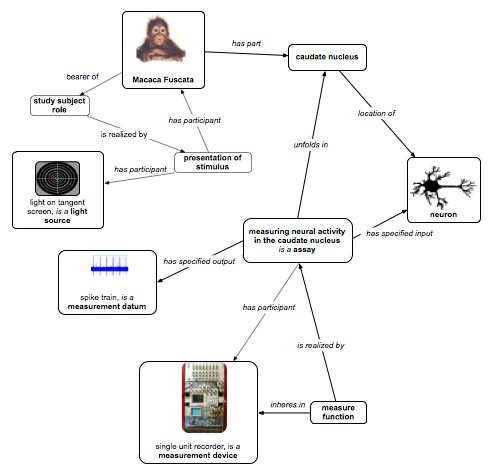
**OBI modeling of a single trial in the neuroscience study (a fragment).** In this and subsequent figures, boxes represent instances, labeled by the class they are instance of and relationships as links labeled in italics. In several cases the parent class is also noted with the class label. Note that in typical use only some instances would be explicitly created – others would be inferred as a consequent of OBI’s definitions. Some processes in this experimental trial are presentation of stimulus, measuring neural activity in the caudate nucleus, and stimulating monkey with light source. Some continuants are *Macaca fuscata*, study subject role, spike train

This investigation studied the role of the primate caudate nucleus in the expectation of reward following action [14]. While the *caudate nucleus* responds preferentially to eye movements in different directions, the response begins prior to eye movement and is dramatically increased when there is expectation of reward for the preferred direction. Here we represent a single trial in which the visual target, a light, is presented to the animal and the neural response is recorded as data. This single trial model contains two processes (Figure 1):

1. Stimulating monkey with a *light source,* which is an example of* presentation of stimulus,* having the participants Japanese macaque monkey as the subject and* light source* as the stimulus, during the process of a *measuring neural activity in the caudate nucleus* assay.

2. *Measuring neural activity in the caudate nucleus:* this process is a subclass of the process *extracellular electrophysiology recording,* which *unfolds in* the *caudate nucleus* that is *part of* the *Macaca fuscata*, of which the Japanese macaque monkey is an example. The anatomical term *caudate nucleus* is imported from the Neuroscience Information Framework standardized (NIFSTD) ontology [15] and used in the logical definition of the assay.

The light on the tangent screen here* is a light source* used to present the stimulus to the study subject. The function of the *microelectrode*, part of the single unit recorder (an example of *processed material*), is realized in the *measuring neural activity in the caudate nucleus* process. The process *measuring neural activity in the caudate nucleus has* the *specified input* a *neuron* and the* specified output* a neuronal *spike train datum*.

## Use case 2: vaccine protection investigation

**Figure 2:**
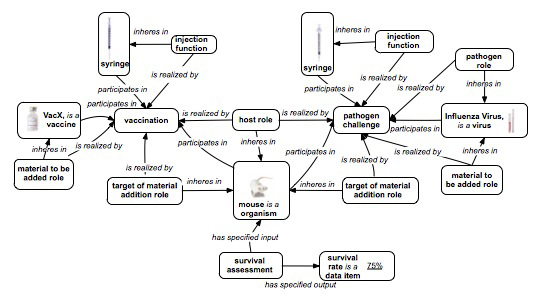
**OBI modeling of vaccine protection investigation (a fragment).** Major processes are vaccination and pathogen challenge, both of which are subtypes of administering substance *in vivo*. The roles target of material addition and material to be added role are defined with respect to this parent class. Some objects are syringe, mouse, host role, target of material addition role, VacX and a portion of Influenza Virus. Note that while the figure shows a single input for the survival assessment, in fact there would be many replicates of the experiment shown, with observations of mouse survival from all of them input to the survival assessment.

A vaccine protection investigation (also known as a vaccine challenge experiment) measures how efficiently a vaccine or vaccine candidate induces protection against a virulent pathogen infection *in vivo*. Figure 2 demonstrates how to use OBI to represent a typical vaccine protection investigation via the following three sub-processes:

1. A* vaccination* is a kind of *administering substance in vivo* process that realizes some *material to be added role,* borne by a *vaccine* (e.g., VacX) as well as a *target of material role* borne by an *organism* that also bears a *host role* (e.g., mouse). The term *vaccination* is a term imported from the Vaccine Ontology (VO, http://www.violinet.org/vaccineontology). An *injection function* that *inheres in* a *syringe* (is a *processed material*) *is realized by* the *vaccination* process.

2. A *pathogen challenge* is also a kind of *administering substance in vivo* process. It realizes a number of roles - a* pathogen role* and *material to be added role* borne by the challenge *organism* (e.g., Influenza Virus), and a *target of material role* and *host role* borne by another *organism* (e.g., mouse). An *injection function* that *inheres in* a *syringeis realized by* the *pathogen challenge* process.

3. A *survival assessment* is an *assay* that measures the *survival rate* (occurrence of death events) in one or more *organisms* that are monitored over time. The *survival assessment* is a protection efficiency assay that *has specified input* a number of *organisms* (e.g., mouse) and *has specified output* a *survival rate*, in this case a *measurement datum* that records that 75% of mice survived the *pathogen challenge*.

## Use case 3: an automated functional genomics investigation

**Figure 3:**
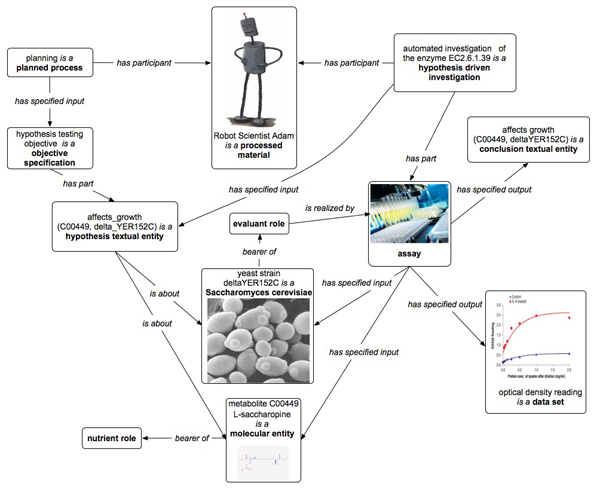
**OBI modeling of the Robot Scientist automated investigation (a fragment).** Major processes are planning, automated investigation of the enzyme EC2.6.1.39, and the assay, while important objects are the Robot Scientist Adam, an amount of yeast strain deltaYER152C, L-saccharopine, the optical density reading data set.

The Robot Scientist “Adam” is designed to perform high-throughput growth curve measurements (phenotypes) of selected microbial strains (genotypes) in a defined media (environment) [7]. The robot requires a complete and precise description of all experimental actions, and this use case demonstrates how OBI can be used to provide elements of such a description. Here we have represented an investigation in which Adam tests hypotheses about which metabolites can restore a function of the removed yeast gene (Figure 3).

1. Adam’s *planning* yields a *plan specification* that has an *objective specification* to test an inferred statements each of which are modeled as a *hypothesis textual entity*. Each statement *is about* whether a particular metabolite will affect yeast strain growth. Adam’s plan specifies an *assay* to test these hypotheses: to grow yeast with and without addition of the metabolite.

2. The *planned process* of the automated investigation of the enzyme EC.6.1.39 is an *instance of* the class *hypothesis driven investigation* with the objective to test the hypothesis specified in the *planning* process (we represent here only a single *hypothesis textual entity* which serves as a design pattern). The result is whether the metabolite affected the growth of the yeast strain (see Figure 3, optical density reading box). The upper growth curve (drawn in red) shows the growth rate with the addition of the metabolite, and the lower curve (drawn in blue) shows the growth rate with no metabolite. So, the addition of the metabolite affects the growth rate of the yeast. The results interpretation is modeled as a *conclusion textual entity* that states that the hypothesis inferred by Adam has been confirmed and the robot can update its background knowledge.

3. The *investigation* process has several *assays* that provide data used to test the hypotheses. The assay *has specified inputs* the metabolite and the yeast strain specified in the *hypothesis*, and the *specified output* is a *data set* consisting of several optical density measurements. The yeast bears the *evaluant role*, and the metabolite the *nutrient role*.

## Discussion

OBI was built to provide a comprehensive and versatile representation of biomedical investigations. Our three biological use cases are represented by statements in terms defined in OBI (see Tables 1 and 2). Individual experimental steps - the two processes in the neuroscience use case, the three processes in the vaccine protection case, and the three processes in the functional genomics case - all fall under *planned process* in OBI.

**Table 1:** Ontology classes used in the three use cases (note: instances are not included):

Classes	Sources and term IDs	Parent class	Use cases
administering substance in vivo	OBI: OBI_0600007	material combination	2
assay	OBI: OBI_0000070	planned process	1,3
caudate nucleus	NeuroLex: birnlex_1373	anatomical entity	1
conclusion textual entity	IAO: IAO_0000144	textual entity	3
data set	IAO: IAO_0000100	data item	3
evaluant role	OBI: OBI_0000067	role	3
extracellular electrophysiology recording	OBI: OBI_0000454	assay	1
function	snap#Function	realizable entity	1,2
host role	OBI: OBI_0000725	role	2
hypothesis driven investigation	OBI: OBI_0000355	planned process	3
hypothesis textual entity	IAO: IAO_0000415	textual entity	3
independent continuant	snap#IndependentContinuant	continuant	
injection function	OBI:OBI_0005246	function	2
interpreting data	OBI: OBI_0000338	process	3
investigation	OBI: OBI_0000066	planned process	3
light source	OBI: OBI_0400065	processed material	1
Macaca fuscata	NCBI_Taxon: NCBITaxon_9542	organism	1
material combination	OBI: OBI_0000652	planned process	2
material to be added role	OBI: OBI_0000319	role	2
material entity	snap#MaterialEntity	Independent continuant	1,2,3
measure function	OBI: OBI_0000453	function	1
measurement device	OBI: OBI_0000832	processed material	1
measurement datum	IAO: IAO_0000109	data item	1,2
measuring neural activity in the caudate nucleus	OBI: OBI_0000812	extracellular electrophysiology recording	1
micro electrode	OBI: OBI_0000816	processed material	1
neuron	FMA: FMA:54527	anatomical entity	1
objective specification	IAO: IAO_0000005	directive information entity	3
organism	OBI: OBI_0100026	material_entity	2
pathogen challenge	OBI: OBI_0000712	administering substance in vivo	2
pathogen role	OBI: OBI_0000718	role	2
plan specification	IAO: IAO_0000104	directive information entity	3
planning	OBI: OBI_0000339	planned process	3
presentation of stimulus	OBI: OBI_0000807	process	1
process	span#Process	processual entity	1,2,3
processed material	OBI: OBI_0000047	material entity	1,2,3
role	snap#Role	realizable entity	1,2,3
Saccharomyces cerevisiae	NCBI_Taxon: NCBITaxon_4932	organism	3
spike train datum	OBI: OBI_0000801	measurement datum	1
study subject role	OBI: OBI_0000097	role	1
survival assessment	OBI: OBI_0000699	assay	2
survival rate	OBI: OBI_0000789	measurement datum	2
syringe	OBI: OBI_0000422	processed material	2
target of material addition role	OBI: OBI_0000444	role	2
vaccination	VO: VO_0000002	administering substance in vivo	2
vaccine	VO: VO_0000001	material entity	2

**Table 2:** Relations used in the three use cases:

Property terms	Sources	Use cases
bearer_of	RO: OBO_REL#bearer_of	1,3
has_part	ro.owl#has_part	3
has_participant	ro.owl#has_participant	1
has_specified_input	OBI: OBI_0000293	1,2,3
has_specified_output	OBI: OBI_0000299	1,2,3
inheres_in	RO: OBO_REL#inheres_in	1,2,3
is about	IAO: IAO_0000136	3
is_a	RO: OBO_REL:is_a	1,2,3
is_realized_by	IAO: IAO_0000122	1, 2, 3
location_of	ro.owl#location_of	1
part_of	ro.owl#part_of	1
participates_in	ro.owl#participates_in	3
unfolds_in	RO: OBO_REL#unfolds_in	1

In the example of the neuroscience investigation use case, the construction of logical definitions of the experimental process encouraged us to ask questions of domain experts because details we wished to capture were not explicit in the publication. For example, was the location of the *micro-electrode* extra- or intra- cellular? Were all *spike train data* recorded from the *caudate nucleus*? How does a spike train relate to the GO biological process *regulation of action potential* [GO:0001508]? Based on the answers, we augmented OBI’s existing assays and imported several terms from external ontologies, for example NIFSTD. When we needed relations that were not yet present in OBI, rather than define them ourselves we used relations from ro_proposed (http://obofoundry.org/cgi-bin/detail.cgi?ro_proposed). For example *unfolds in* specifies that an occurrent (process) happens in a certain location (i.e., the assay of spike trains in the caudate nucleus). Finally, we used the NCBI taxonomy [16] to describe the species involved in this experiment. Re-use of external resources fulfils two purposes. First, as domain experts have already devoted time to defining terms in these external ontologies we save ourselves substantial efforts by not replicating that work. Second, by re-using existing resources that others already use, we improve the potential for future data integration by making it unnecessary to map between different identifiers denoting the same entity.

In developing the neuroscience use case we found decisions about choosing an appropriate level of detail challenging: in this use case we decided not to include instances of the classes, but instead to focus on adding classes that can be re-used for other use cases and communities. It is our intention that our analysis and the classes we defined serve as design patterns for other neuroscience assays. Depending on the use case, OBI intends to be able to model the desired level of details (granularity), from molecular level experiments to higher level of biomedical investigations. OBI can be used at a more or less granular level depending on the user community needs.

In the second use case, the vaccine protection investigation includes three processes. The processes *vaccination* and *pathogen challenge* are disjoint subclasses of *administering substance in vivo*. The process *Survival assessment* is a type of *assay* (Table 1). We found that all these required processes, as well as all other entities described in the use case could be represented using OBI idioms. *Syringe* is a *processed material* that *participates in* different processes. Entities such as *vaccine* are types of *material entity*. *Host role*, *pathogen role*, and *material to be added role* are types of *role*.

That OBI can be used to represent experimental processes for different applications and domains is appealing because it suggests that we can better leverage the work we each do. For the domain of vaccine investigation, approximately 400 vaccines have been manually curated and stored in the Vaccine Investigation and Online Information Network (VIOLIN; http://www.violinet.org) vaccine database system [17]. Currently, the vaccine protection experimental data in VIOLIN is stored in plain text and can be difficult to interpret. The lack of a common ontology to aid in representing this data has prevented optimal use of the VIOLIN vaccine data. We plan to apply the representation described in this paper to that data in order to enable advanced querying both within the data as well as across data from other biomedical communities that represent their data using OBI. As an example, consider that a vaccine candidate against Alzheimer disease may induce specific changes on the brains of transgenic mice or human patients (http://www.ncbi.nlm.nih.gov/pubmed/12379846). Therefore enabling queries across the domains of vaccinology and neuroscience would be of utility in conducting such research.

The representations of the investigations run by Adam were stored as instances of the defined classes in a relational database [18]. Accurate and complete recording of all experimental processes involved in the investigations allows efficient re-use of produced data and results for different investigations with different objectives. OBI’s approach to representation for automation suggests new possibilities for automated investigations, desirable because such methods offer high throughput mechanisms not only for data generation, but also for hypotheses generation and the results analysis. As using terminology from a wide range of biology is a central part of OBI’s methodology, we can easily imagine that it is reasonable to extend the reach of such an approach. For example, DNA microarray experiments may also be performed using Robot Scientists in order to generate and test hypotheses regarding the transcript expression level in brain or other tissues, and knowledge encapsulated the Gene Ontology or other ontologies could be applied to interpreting the results of such experiments.

## Conclusions

Here we provide three real world use cases as examples of how to represent experimental processes with OBI. Experience such as this helps validate OBI’s current design choices, as well show how to extend it in domain specific ways. It also generates competency questions that allow us to identify parts of OBI that are insufficiently expressive and to identify external resources that can be used to extend OBI’s coverage. We found that a major technical challenge is the requirement to import terms from other ontologies to construct logical definitions: due to its broad scope OBI spans multiple existing ontological resources. There is a significant cost preventing those large imports, as reasoning becomes slower and the ontology is harder to navigate. To solve this problem the OBI consortium developed the MIREOT mechanism [19], which preserves namespaces of imported terms and allows their direct use into OBI. We also hope that technologies such as views [20] and modules [21] as well as improvements to existing reasoners will address these issues. OBI will be further developed to expand the coverage and depth of biomedical investigations and the use cases presented here helped us in testing the version 1.0 of the ontology.

## List of abbreviations used

EXPO: EXPeriment Ontology; GO: Gene Ontology; IAO: Information Artifact Ontology; LABORS: LABoratory Ontology for Robot Scientists; MGED: Microarray Gene Expression Society; MIREOT: Minimum Information to Reference an External Ontology Term; MSI: Metabolomics Standards Initiative; NCBI: National Center for Biotechnology Information; NCBI_Taxon: NCBI Taxonomy; NIFSTD: Neuroscience Information Framework standardized; OBI: Ontology for Biomedical Investigations; OBO: Open Biomedical Ontologies; VIOLIN: Vaccine Investigation and Online Information Network; VO: Vaccine Ontology.

## Competing interests

The authors declare that they have no competing interests.

## Authors' contributions

The three use cases were primarily provided by DD/JT, YH, and LNS. All authors contributed to the development of OBI.
